# Supplementary material for: Single-cell epigenomics and spatiotemporal transcriptomics reveal human cerebellar development
Source: Nat Commun. 2023 Nov 22;14:7613. doi: 10.1038/s41467-023-43568-6 (PMC10665552; doi:10.1038/s41467-023-43568-6)
Supplement: Supplementary file 1 — Supplementary Information [file 41467_2023_43568_MOESM1_ESM.pdf]

Supplementary Information

Single-cell epigenomics and spatiotemporal transcriptomics reveal human cerebellar development

Suijuan Zhong[1,2,6,#,*], Mengdi Wang[3,4,#], Luwei Huang[3,4,#],Youqiao Chen[1,#], Yuxin Ge[1], Jiyao Zhang[1], Yingchao Shi[5], Hao Dong[3,4], Xin Zhou[1,2,6], Bosong Wang[1], Tian Lu[3,4], Xiaoxi Jing[1], Yufeng Lu[3], Junjing Zhang[1], Xiaoqun Wang[1,2,6], Qian Wu[1,2,6,*]

[1]State Key Laboratory of Cognitive Neuroscience and Learning, New Cornerstone Science Laboratory, Beijing Normal University, Beijing, 100875, China

[2]IDG/McGovern Institute for Brain Research, Beijing Normal University, Beijing, 100875, China

[3]State Key Laboratory of Brain and Cognitive Science, Institute of Biophysics, Chinese Academy of Sciences, Beijing 100101, China

[4]University of Chinese Academy of Sciences, Beijing, 100049, China

[5]Guangdong Institute of Intelligence Science and Technology, Guangdong 519031, China

[6]Changping Laboratory, Beijing 102206, China

[#]These authors contributed equally

*Corresponding authors: Qian Wu (qianwu@bnu.edu.cn) and Suijuan Zhong (zhongsuijuan@bnu.edu.cn)

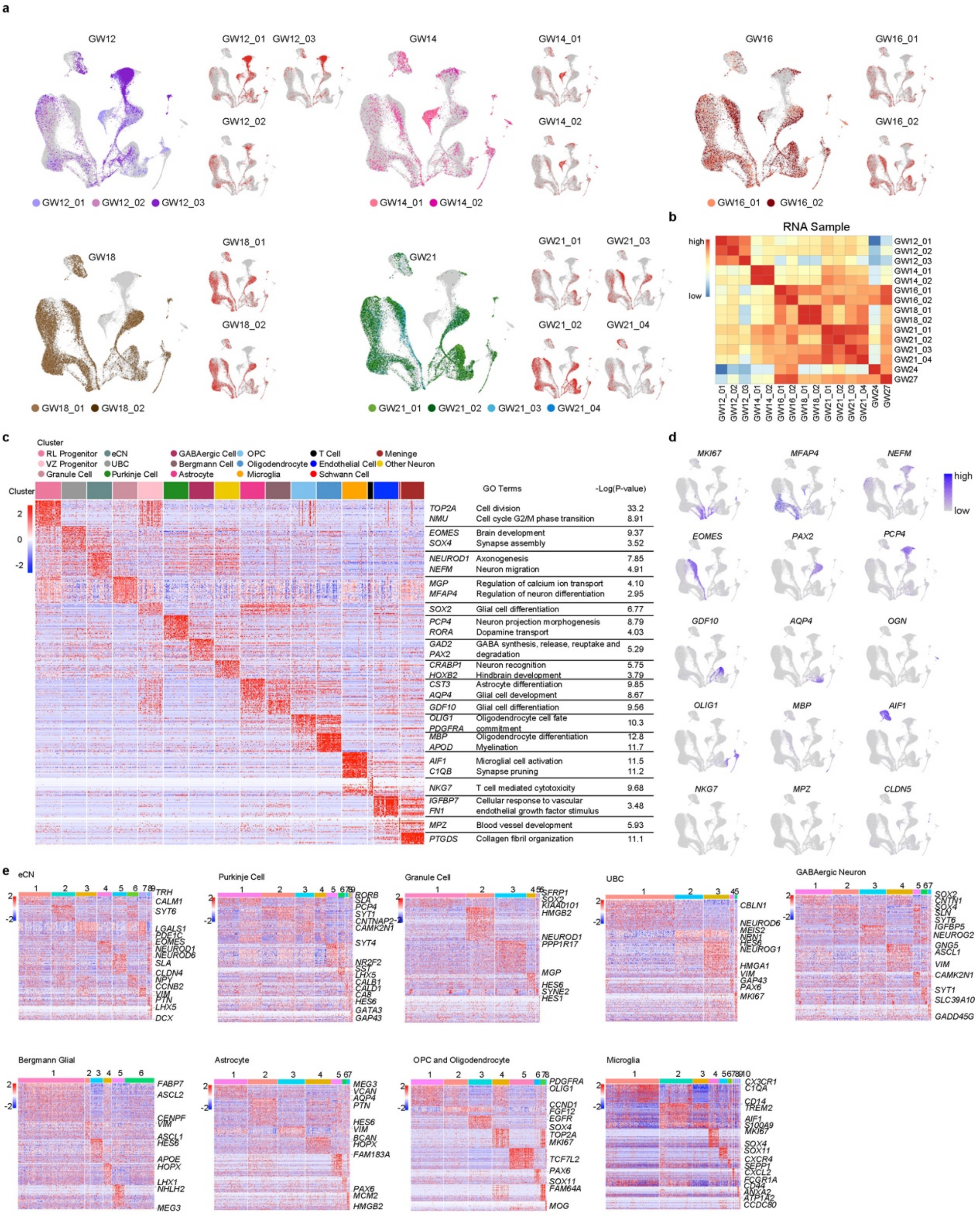

Supplementary Figure 1 | Sample information of single-cell RNA-seq in the developing human cerebellum, related to Figure 1. a, UMAP plots of cells in the cerebellum. Repetitions of GW12, GW14, GW16, GW18 and GW21 are labeled in different colors, and no obvious distribution differences were observed among the different batches from the same embryo stages. Each cell color represents the gestational week. **b,** Correlation of samples of RNA-seq showing the similarity of samples. **c,** Heatmap showing the expression level and identity of genes in all cells in the developing cerebellum in RNA-seq. **d,** The expression of known markers is shown using the same layout as in Fig. 1A (RNA). **e,** Heatmaps show the subclasses of eCN, Purkinje Cell, Granule Cell, UBC, GABAergic Neuron, Bergmann Glial, Astrocyte, OPC, Oligodendrocyte and Microglia. The genes

were organized into clusters. The bar chart on the top shows the clusters. Specific genes related to each subtype are highlighted on the right.

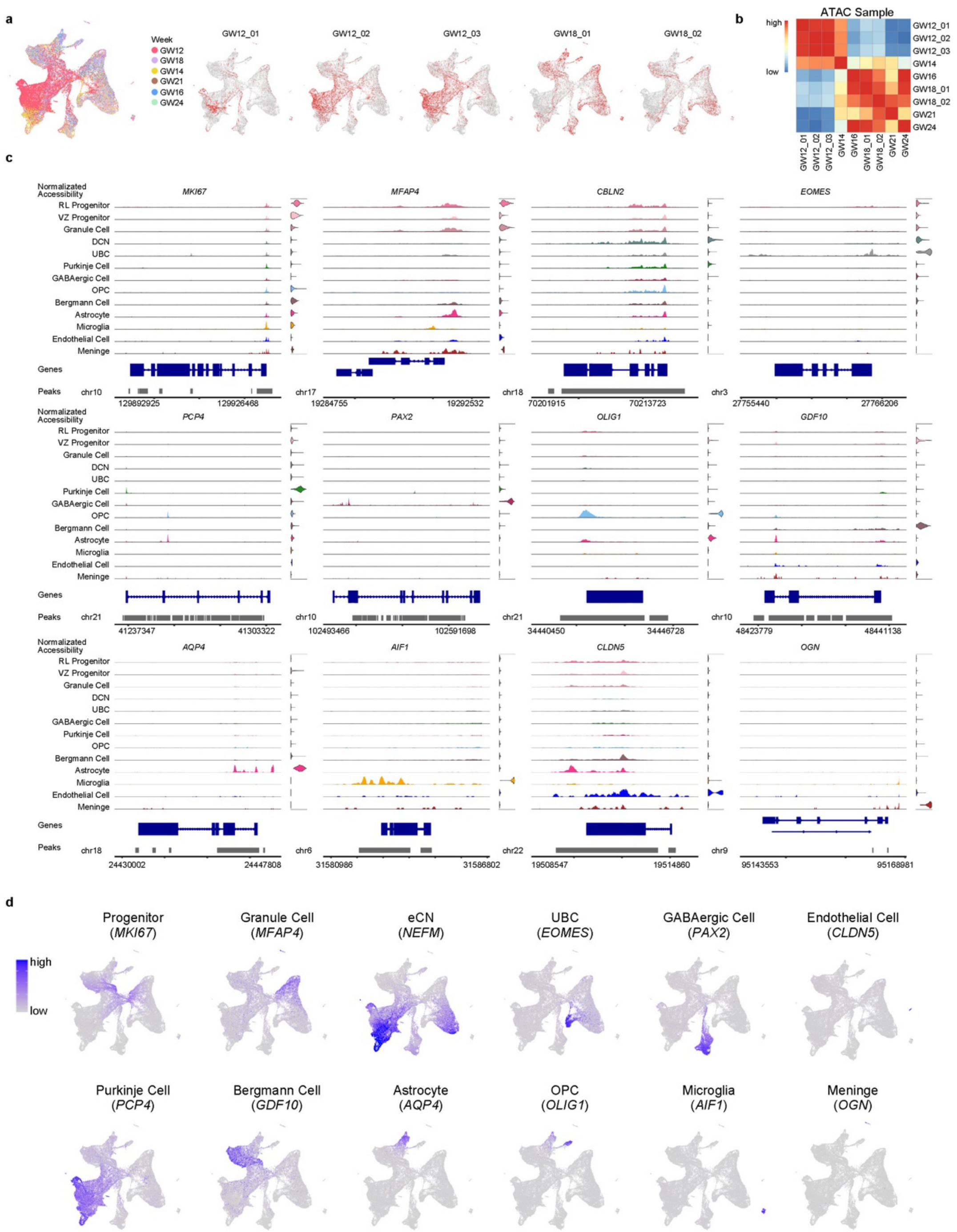

Supplementary Figure 2 | Sample information of single-cell ATAC-seq in the developing human cerebellum, related to Figure 1. a, UMAP plots of cells in the cerebellum. Each cell color represents the gestational week. Repetitions of GW12 and GW18 are labeled, and no obvious distribution differences were observed among the different batches from the same embryo stages. Each cell color represents the gestational week. **b,** Correlation of samples of ATAC-seq showing the similarity of samples. **c,** Pseudobulk peak plots showing the activation level and identity of genes in all cells in the developing cerebellum in ATAC-seq. Vln plots on the right show the expression level of the genes. **d,** The expression of known markers is shown using the same layout as in Fig. 1B (ATAC); gray, no expression; blue, relative expression.

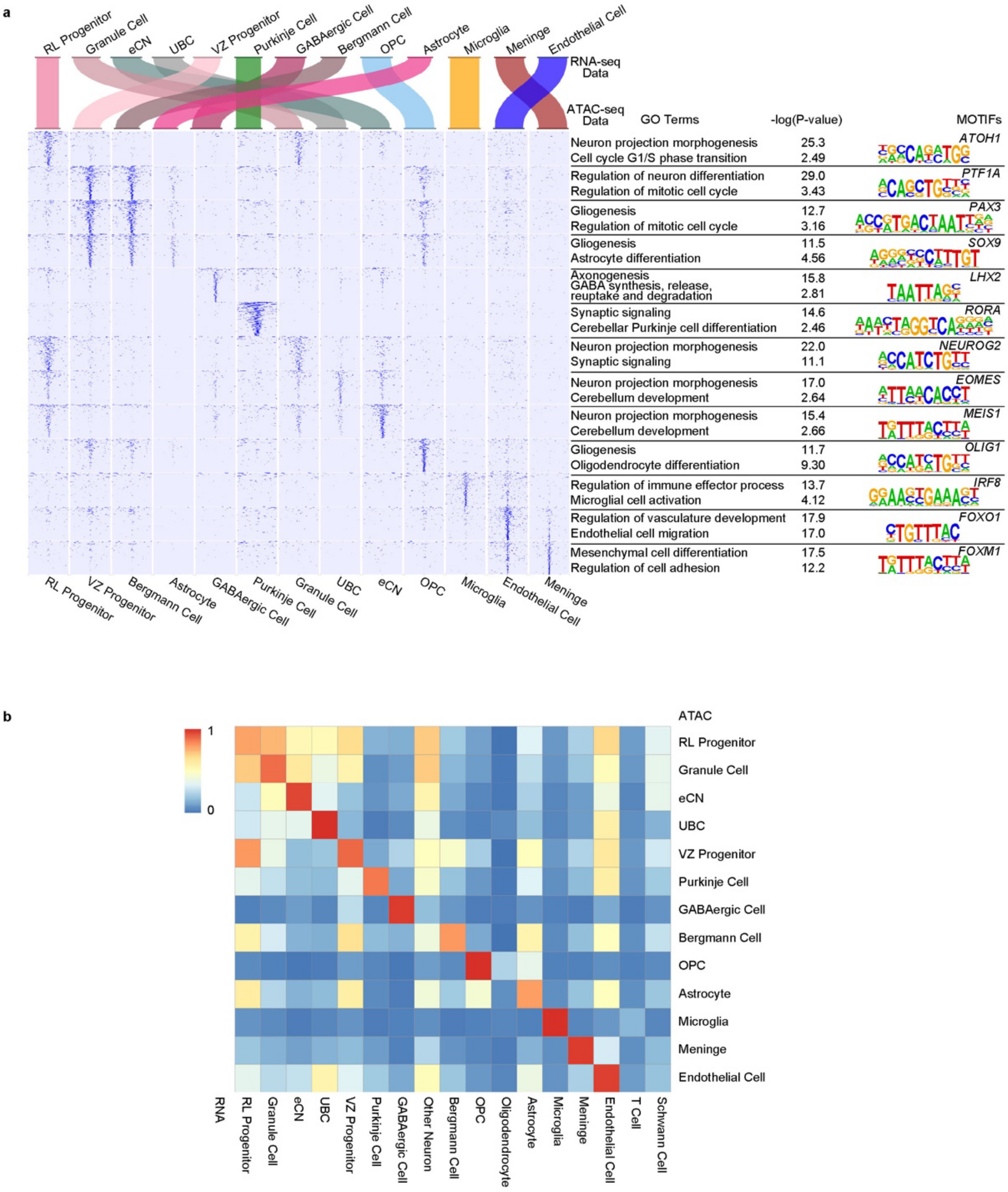

Supplementary Figure 3 | Correlations of single-cell RNA-seq and ATAC-seq in the developing human cerebellum, related to Figure 1. a, Heatmap of scATAC cluster top 1000 DA peaks. Pileups are centered on peak centers, and the +/-20 kb flanking region is depicted. Top, Sankey plot showing the correlation of scATAC-seq clusters and scRNA-seq clusters. Right panel, GO terms and significantly enriched TF motifs for each cluster-specific peak set as determined by HOMER. **b,** Heatmaps showing the correlations of RNA-seq and ATAC-seq clusters.

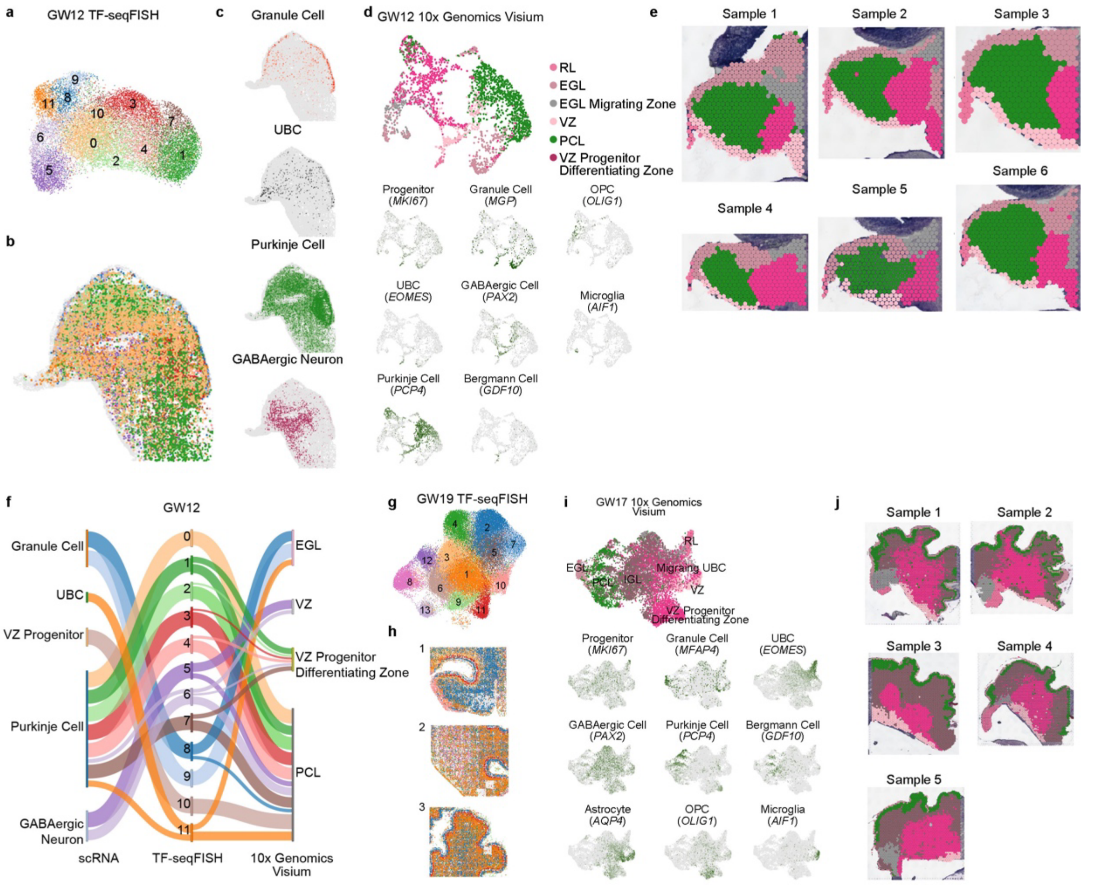

Supplementary Figure 4 | Sample information of spatial patterns in the developing human cerebellum, related to Figure 1. a, UMAP plots showing the cluster information from GW12 TF-seqFISH. **b,** TF-seqFISH plots showing the spatial distribution of clusters in the GW12 cerebellum. **c,** TF-seqFISH plots showing the spatial distribution of different cell types in the GW12 cerebellum. **d,** UMAP plots showing the spatially related clusters and gene expression in the GW12 10x Genomics Visium data. **e,** Repetitions of GW12 10x Genomics Visium data. No obvious distribution differences were observed among the different batches. **f,** Sankey plot showing the correlation among scRNA, TF-seqFISH and 10x Genomics Visium Data in the GW12 human cerebellum. **g,** UMAP plots showing the cluster information of GW19 TF-seqFISH. **h,** TF-seqFISH plots showing the spatial distribution of clusters in the GW19 cerebellum. **i,** UMAP plots showing the spatially related clusters and gene expression in the GW17 10x Genomics Visium data. **j,** Repetitions of GW17 10x Genomics Visium data. No obvious distribution differences were observed among the different batches.

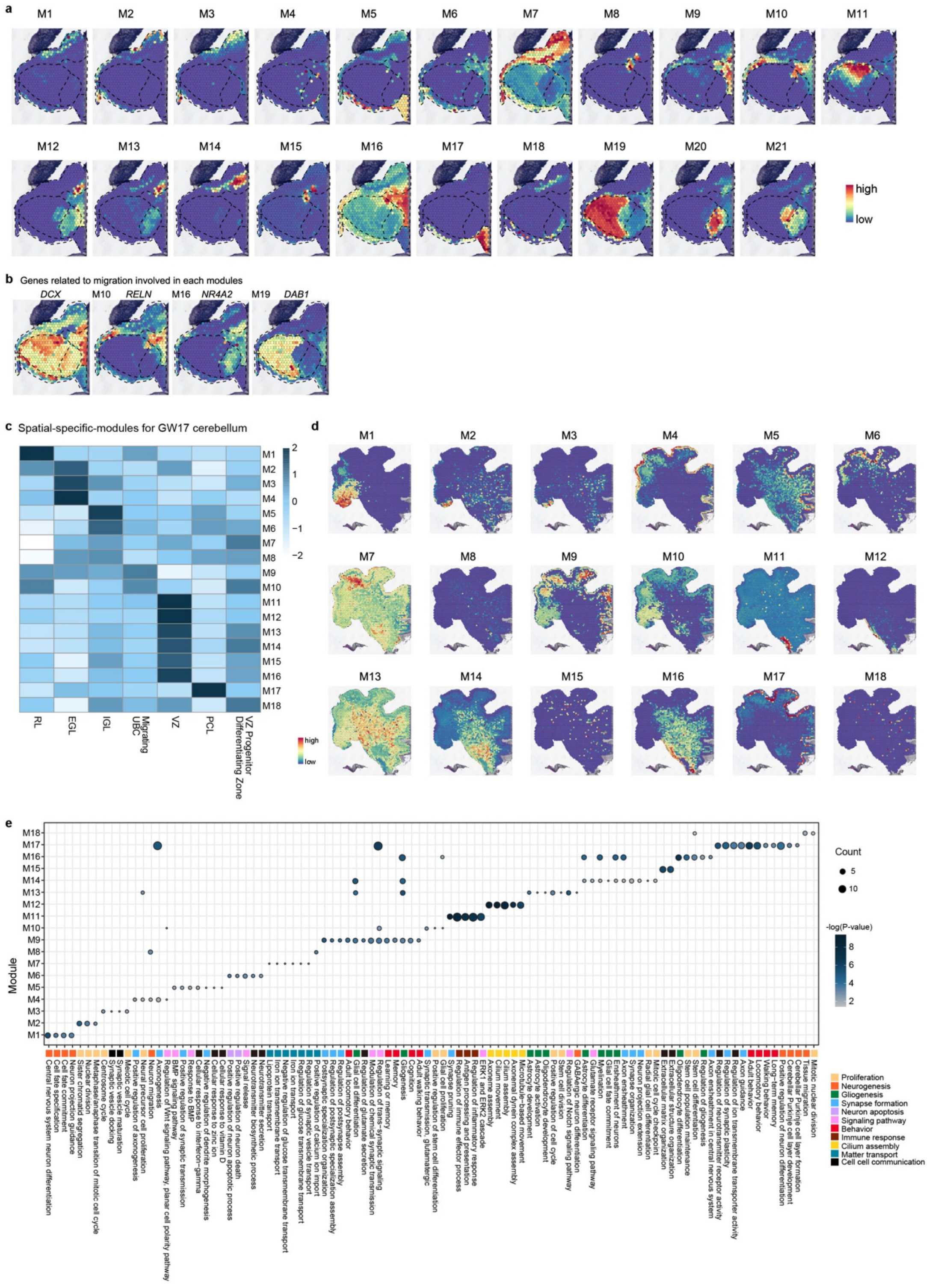

Supplementary Figure 5 | Spatial-specific patterns in the developing human cerebellum, related to Figure 1. a, The gene patterns of each module in the GW12 cerebellum are shown in the same layout in Fig. 1C. **b,** Spatial patterns of genes related to migration involved in each module. **c,** Heatmap showing the spatial-specific modules for the GW17 cerebellum. **d,** The gene patterns of each module in the GW17 cerebellum are shown in the same layout in Fig. 1D. **e,** Gene Ontology analysis of spatial-specific modules showing the KEGG pathways or biological processes in the

GW17 cerebellum. Dots show the numbers of genes in each module, and the scale bar shows the -log(P-value) for the GO terms. Hypergeometric test.

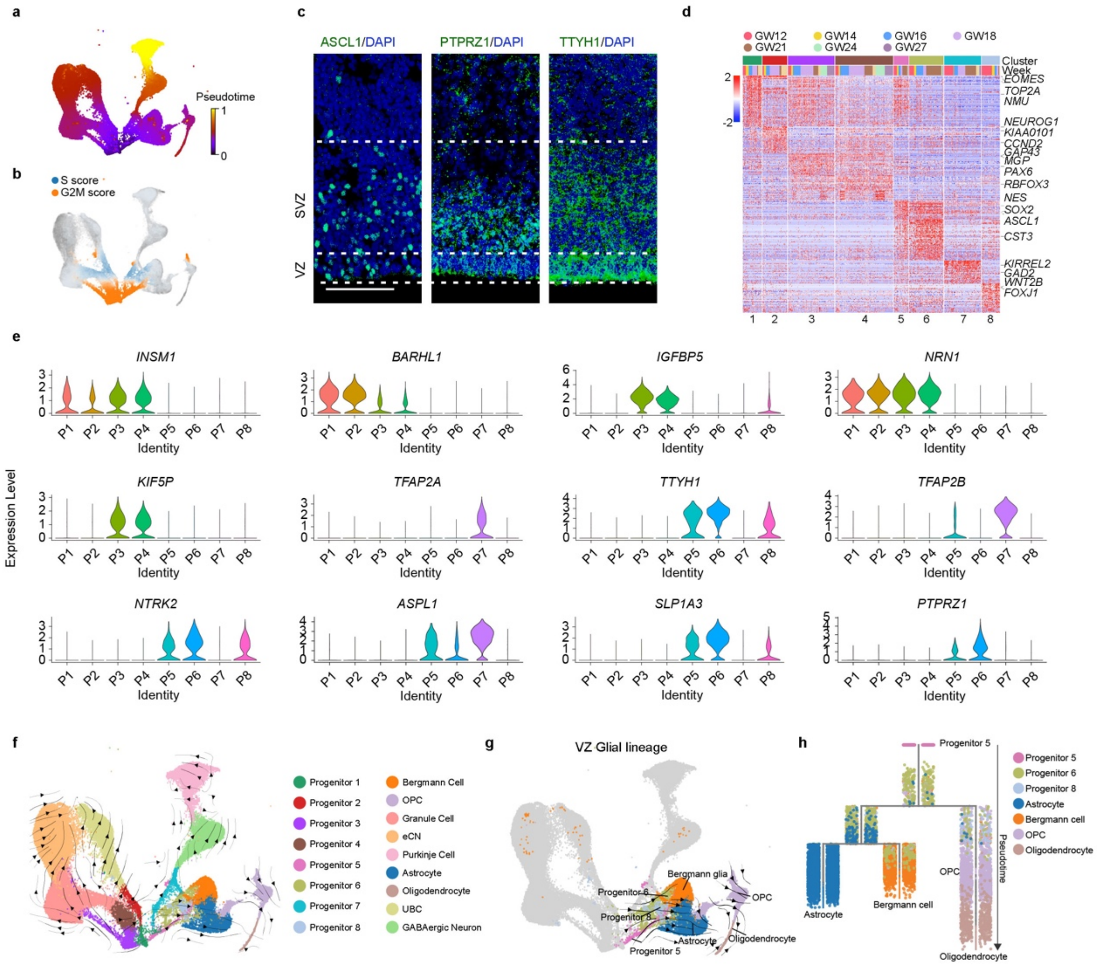

Supplementary Figure 6 | Molecular Diversity of Progenitors in the Cerebellum, related to Figure 2. a, Velocity visualization of the pseudotime information in the developing human cerebellum. **b,** Velocity visualization of the cell cycle information in the developing human cerebellum. **c,** Immunostaining of ASCL1, PTPRZ1 and TTYH1 showing VZ progenitors in the GW10 cerebellum. Scale bar, 500 µm (top); 100 µm (bottom). The experiments were repeated three times independently with similar results. **d,** Heatmap showing the differentially expressed genes in the subgroups in Progenitor. Highly expressed genes are shown on the right. The colored bars on the top show the clusters and weeks. **e,** Vlnplots showing the expression level of the genes in the progenitor subgroups. **f,** Velocity visualization of all cell types using the same layout as in Figure 2a. Each dot represents a single cell, and cells are laid out to show similarities. Each cell color represents the cell type. **g,** Velocity visualization of glial lineage using the same layout as in Figure 2a. Each dot represents a single cell, and cells are laid out to show similarities. Each cell color represents the cell type. **h,** URD plot of glial lineage showing the trajectory information of different glial progenitor subtypes.

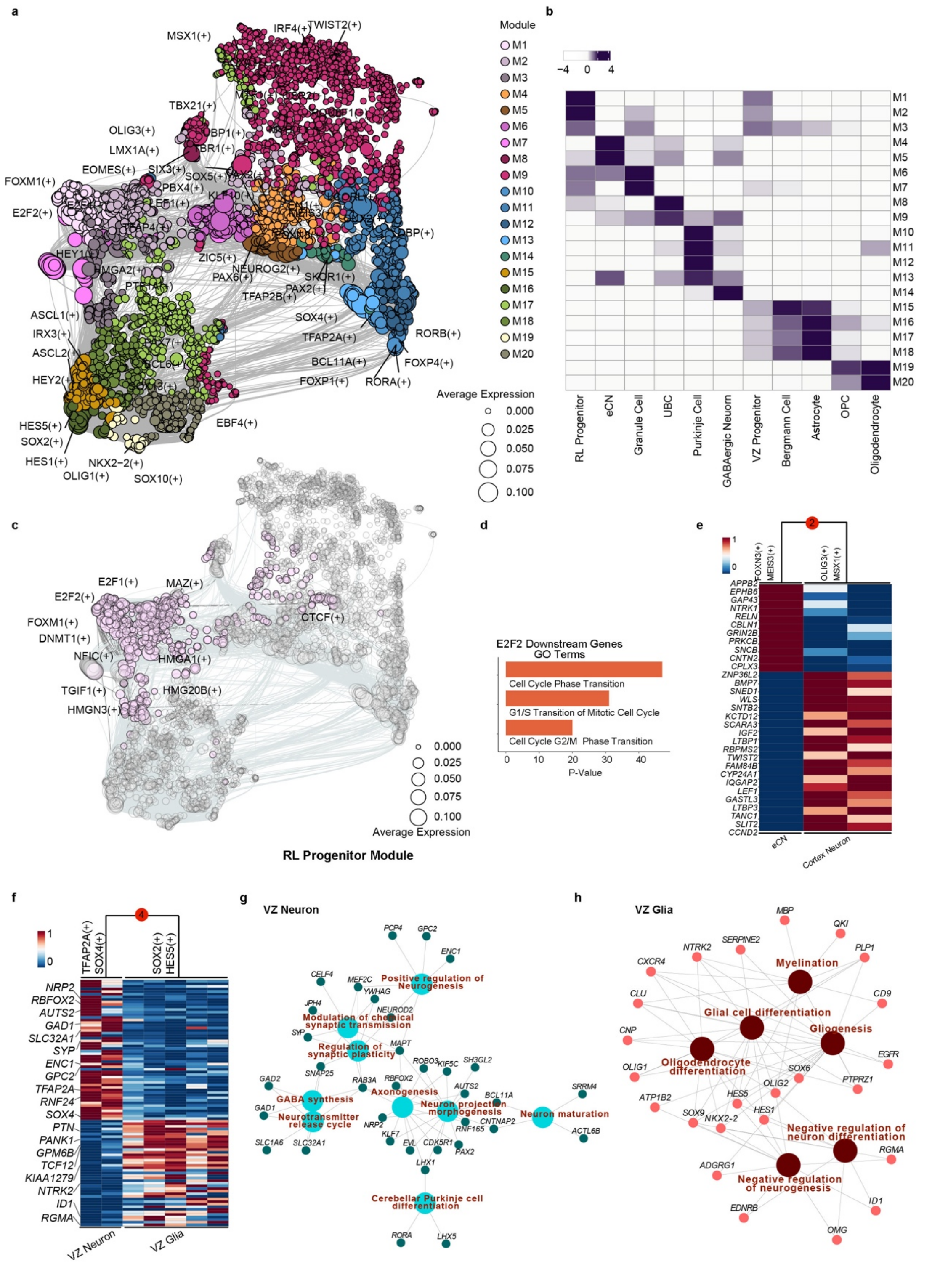

Supplementary Figure 7 | Regulon modules typifying cell type specificity in the developing human cerebellum, related to Figure 2. a, Clusters of regulon-target modules in the developing human cerebellum. **b,** Heatmaps of regulon-target modules showing the gene expression enrichment in each cell type. **c,** RL Progenitor Module showing the key regulons in the maintenance of RL progenitors. **d,** GO terms of the target genes of regulon E2F2. Hypergeometric test. **e,** Heatmap showing the expression level and identity of target genes in eCNs and cortical neurons. **f,** Heatmap

showing the expression level and identity of target genes in VZ neurons and VZ glial cells. Specific gene expression in each type is shown on the left of the heatmap panel. **g-h,** GO terms showing the biological functions of the target genes of regulon. TFAP2A, SOX4 (**g**) and SOX2, HES5 (**h**) in the VZ lineage. Hypergeometric test.

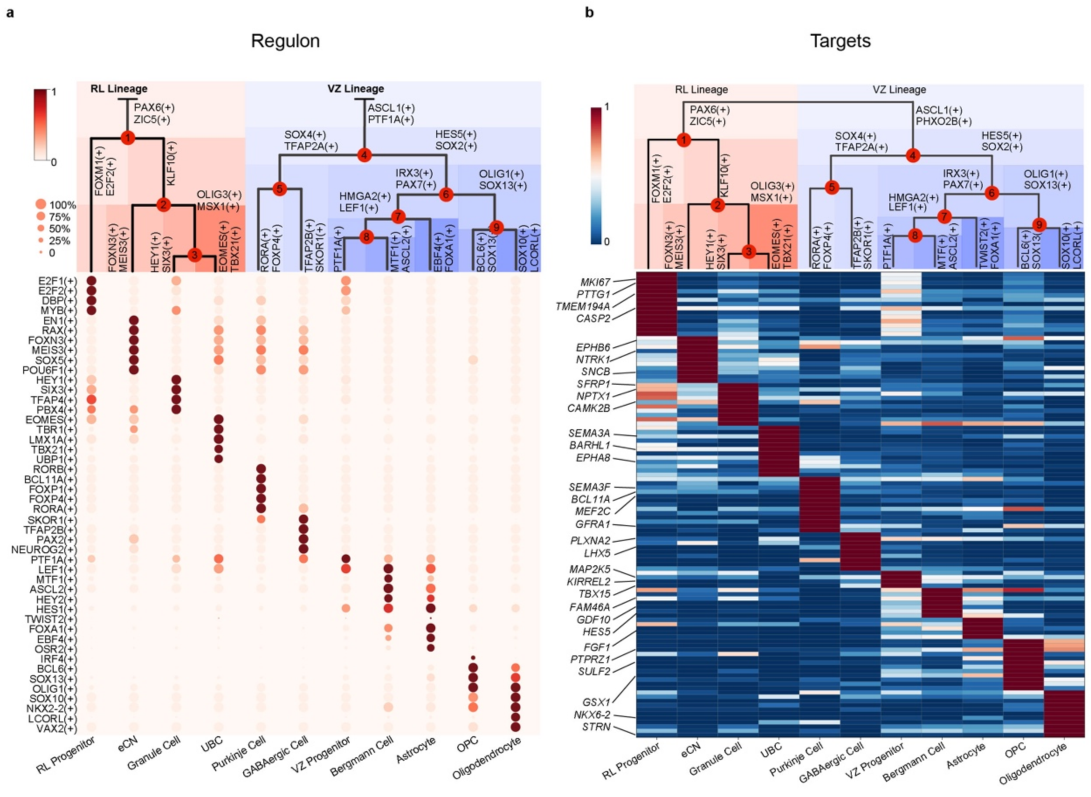

Supplementary Figure 8 | Regulon hierarchical networks in the cerebellum, related to Figure 2. a, A dendrogram of regulons on the top for each cell cluster in the main lineage in the cerebellum except for microglia, meninges, T cells, Schwann cells, endothelial cells and other neurons, showing the lineage trajectory in the developing cerebellum. The TFs shown at each branching of the dendrogram are representative of subjacent groups of regulons. Dot plots at the bottom show the expression level and identity of Regulon in each cell type. **b,** A dendrogram of regulons on the top for each cell cluster in the main lineage in the cerebellum except for microglia, meninges, T cells, Schwann cells, endothelial cells and other neurons, showing the lineage trajectory in the developing cerebellum. The TFs shown at each branching of the dendrogram are representative of subjacent groups of regulons. The heatmap at the bottom shows the expression level and identity of target genes in each cell type. Specific gene expression in each type is shown on the left of the heatmap panel.

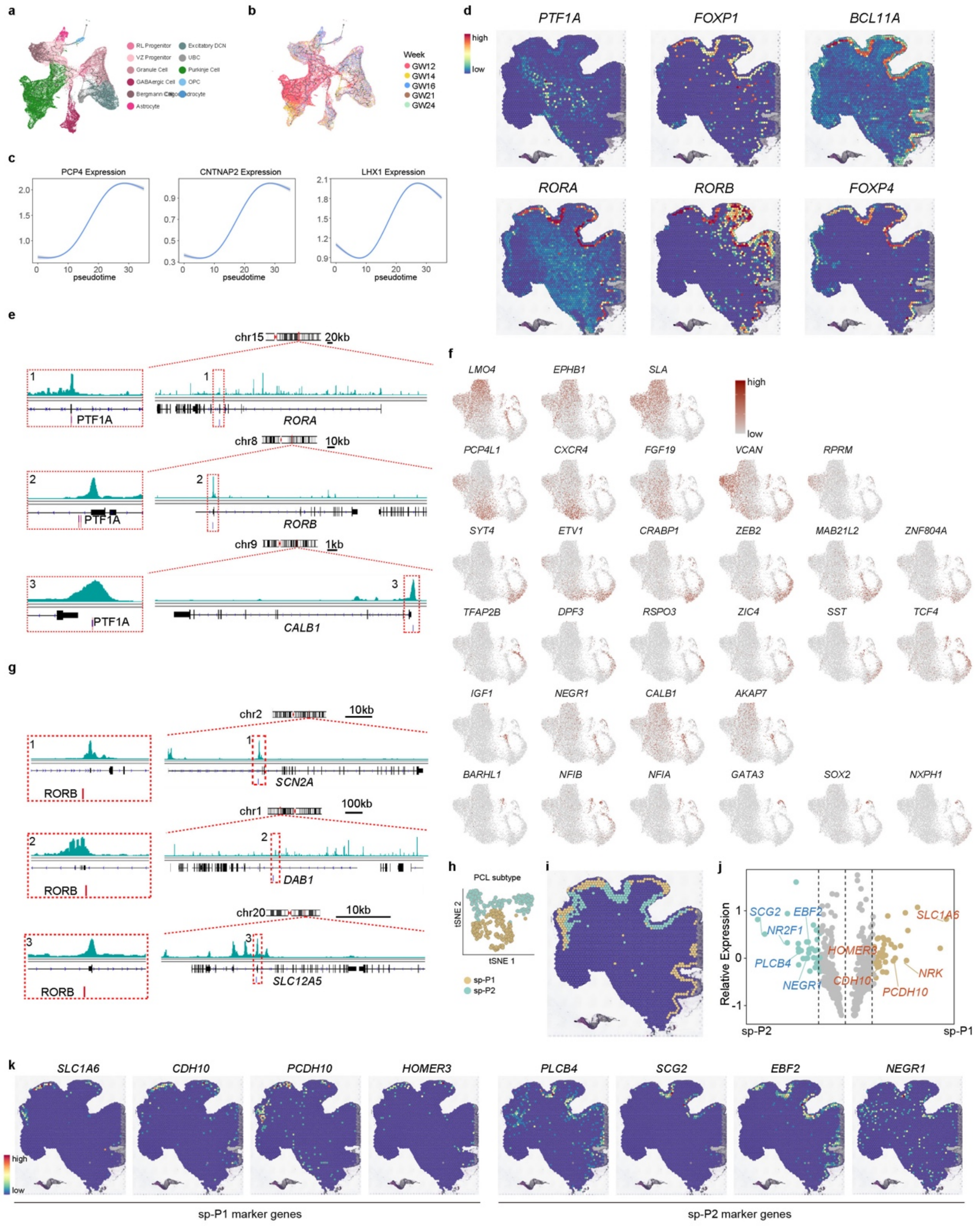

Supplementary Figure 9 | Diversity of neurogenesis of Purkinje cells in the developing human cerebellum, related to Figure 3. a-b, Cell lineage relationships of all cell types except for Meninge and microglia. analyzed in the developing human cerebellum in ATAC-seq using the same layout as in Fig. 1B. Each dot represents a single cell; the color of each cell represents the cell type (**a**) and week (**b**). **c,** Expression dynamics of *PCP4, CNTNAP2* and *LHX1* over pseudotime in VZ progenitor and Purkinje cells. Smoothed *PCP4, CNTNAP2* and *LHX1* expression was normalized by the size factor in each single cell and then log-transformed and scaled, the shadow represents the 95% confidence interval around the fitted curve. **d,** 10x Genomics Visium data showing the expression of different markers in Purkinje cells in the GW17 cerebellum. **e,** Normalized pseudobulk ATAC-

seq profiles of *RORA, RORB* and *CALB1* in the cerebellum showing the activation of regulon PTF1A target genes. The amplifying panel shows the predicted PTF1A binding sites. **f,** The expression of known markers is shown using the same layout as in Fig. 3f; gray, no expression; red, relative expression. **g,** Normalized pseudobulk ATAC-seq profiles of *SCN2A, DAB1* and *SLC12A5* in the cerebellum showing the activation of regulon RORB target genes. The amplifying panel shows the predicted RORB binding sites. **h,** tSNEPlot showing the spatially related subclusters in Purkinje cells in the developing human cerebellum. **i,** 10x Genomics Visium data showing the spatial distribution of two subclusters of Purkinje cells in the GW17 cerebellum. **j,** Scatterplot of all genes correlated with the differentiation network across sp-P1 (gold plot) and sp-P2 (blue plot) in Purkinje cells. **k,** 10x Genomics Visium data showing the expression of different markers in the GW17 cerebellum.

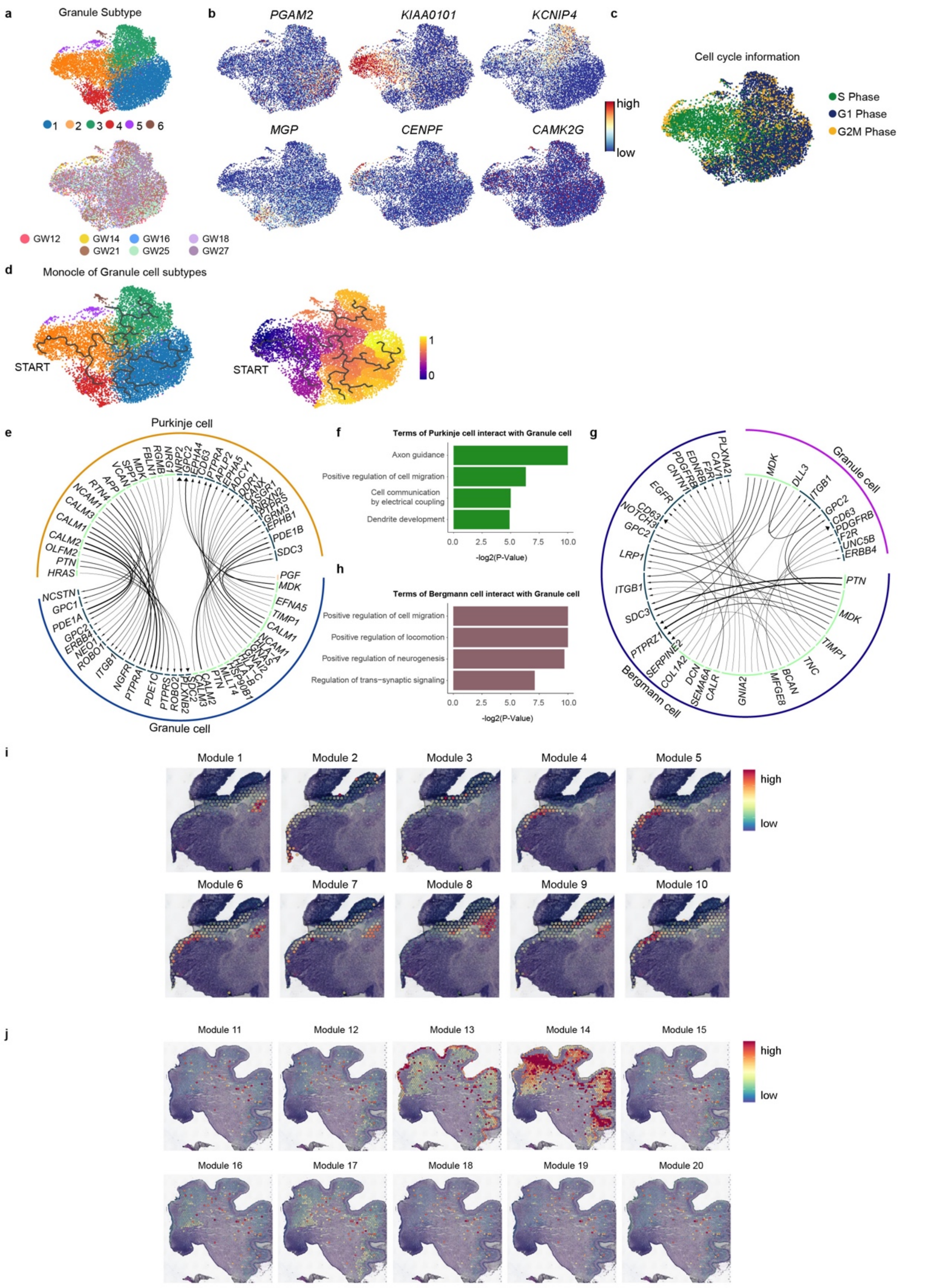

Supplementary Figure 10 | Diversity of neurogenesis of granule cells in the developing human cerebellum, related to Figure 4. a, Visualization of six subclasses of granule cells in the developing human cerebellum using UMAP. Each dot represents a single cell, and cells are laid out to show similarities. Each cell color represents the cell type. **b,** Markers for granule subtypes shown in the UMAP plots, blue, no expression; red, relative expression. **c,** Visualization of cell cycle information of granule cells in the developing human cerebellum using UMAP. **d,** Cell lineage relationships of

6 subtypes in granule cells. Each dot represents a single cell; the color of each cell represents the cell type (left) and pseudotime (right). **e-f,** LR Plot depicting the relationships of ligands and receptors from Purkinje cells and granule cells (**e**) and the GO terms analysis among these ligands and receptors (**f**). Hypergeometric test. **g-h,** LR Plot depicting the relationships of ligands and receptors from bergmann cells and granule cells (**g**) and the GO terms analysis among these ligands and receptors (**h**). Hypergeometric test. **i,** The gene patterns of each module in Fig. 4j show the RL to EGL process in the GW12 cerebellum. **j,** The gene patterns of each module in Fig. 4m show the RL to EGL process in the GW17 cerebellum.

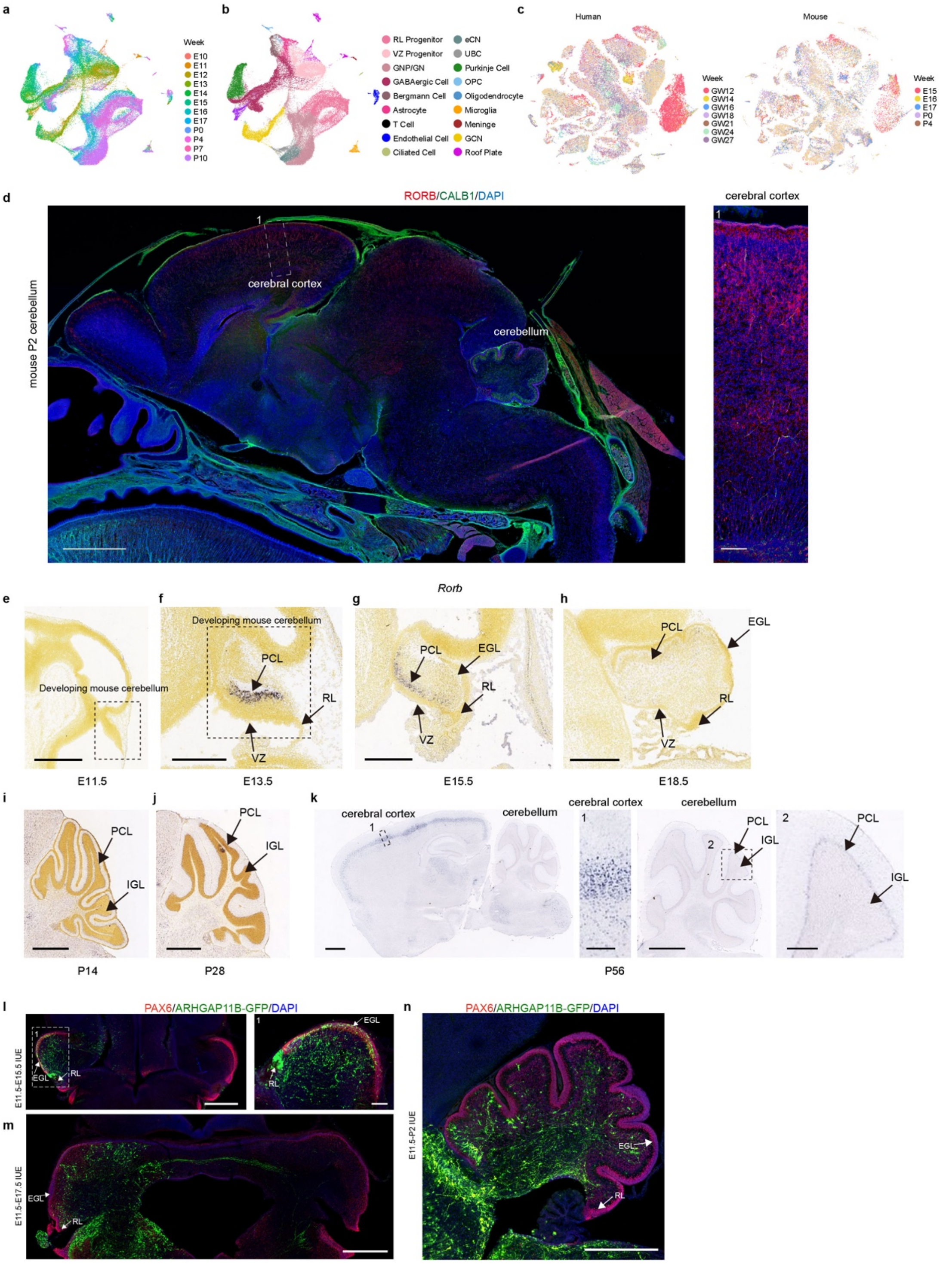

Supplementary Figure 11 | Dynamics of the developing human and mouse cerebellum, related to Figure 5. a-b, Visualization of 18 cell types in the developing mouse cerebellum using UMAP. Each dot represents a single cell, and cells are laid out to show similarities. Each cell color represents the week (**a**) and cell type (**b**). **c,** tSNE visualization of human (GW12-27) and mouse (E15-P4) neural lineages. Cell types were analyzed using CCA. Each cell color represents the week, human (left) and mouse (right). **d,** Immunofluorescence images of RORB and CALB1 in mouse P2. Scale bar, 1000 μm (left), 100 μm (right). The experiments were repeated three times independently with

similar results. **e-h,** In situ hybridization of *RORB* in the developing mouse cerebellum at E11.5 (**e**), E13.5 (**f**), E15.5 (**g**) and E18.5 (**h**). Scale bar, 800 µm (**e**), 400 µm (**f**), 400 µm (**g**), 400 µm (**h**). **i-k,** In situ hybridization of *RORB* in the mouse cerebellum at P14 (**i**), P28 (**j**) and P56 (**k**). Scale bar, 1000 µm (**i**), 1000 µm (**j**), 1000 µm (**k**, left 1), 200 µm (**k**, left 2), 1000 µm (**k**, left 3), 200 µm (right). **l-n,** Overexpression of ARHGAP11B at E11.5 promotes cerebellar cortex folding observed at E15.5 (**l**), 17.5 (**m**) and P2 (**n**) in mice. Scale bars, 500 µm (**l**, left), 100 µm (**l**, right), 500 µm (**m**) and 500 µm (**n**). The experiments were repeated three times independently with similar results.

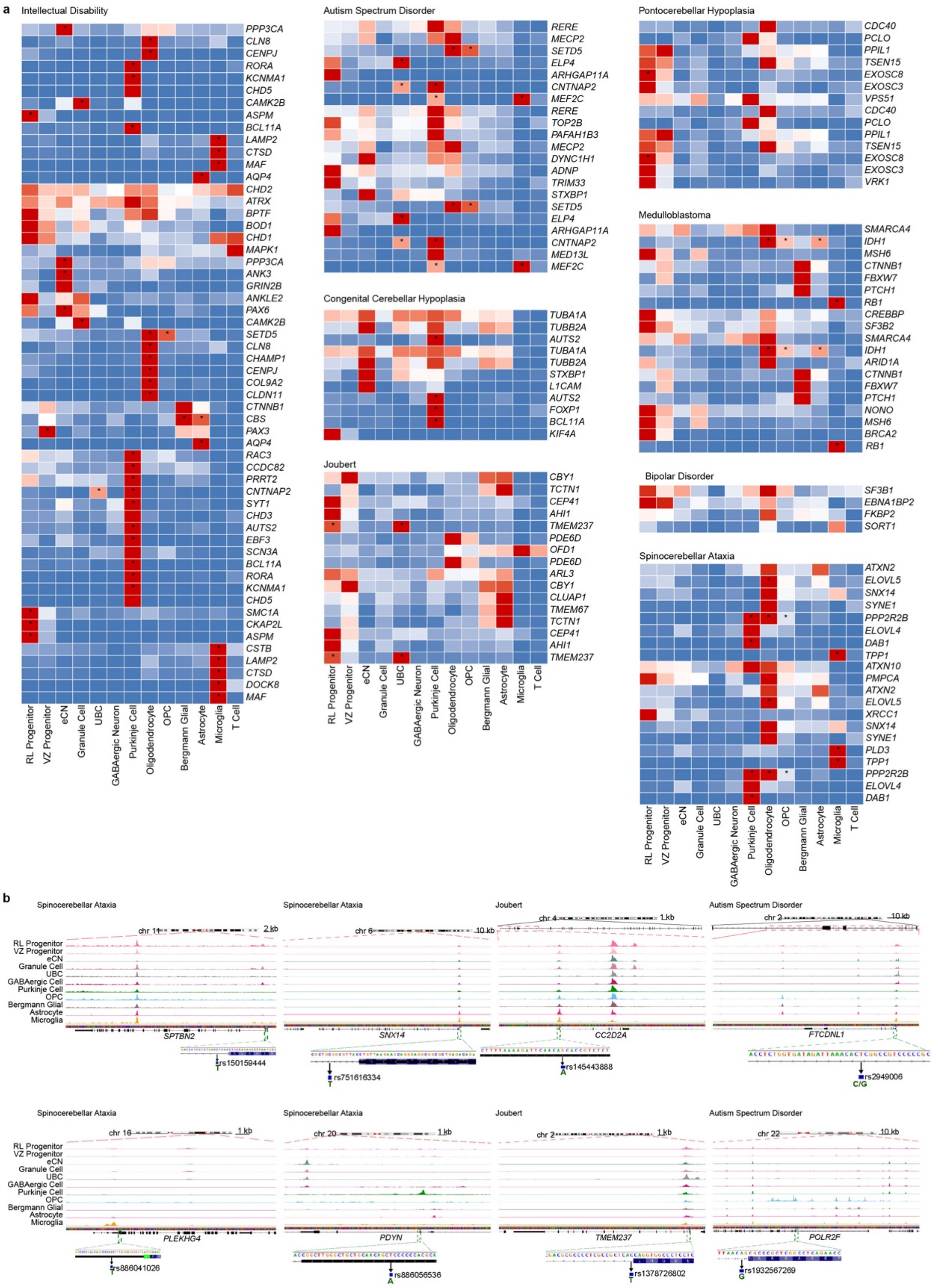

Supplementary Figure 12 | Gene expression of diseases in the human cerebellum, related to Figure 6. a, Expression patterns of selected disease-associated genes in each cell type. “*” represents genes with significantly different expression when compared with cells from other cell types. Two-sided wilcox.test. **b,** Normalized ATAC-seq profiles of SPTBN2, SNX14, PLEKHG4, PDYN, CC2D2A, FTCDNL1, TMEM237 and POLR2F in the cerebellum, with all cell types

showing the activation of these genes. The amplifying panel shows the SNP site in the transcription start site (TTS).
