# Supplementary material for: Single-cell epigenomics and spatiotemporal transcriptomics reveal human cerebellar development
Source: Nat Commun. 2023 Nov 22;14:7613. doi: 10.1038/s41467-023-43568-6 (PMC10665552; doi:10.1038/s41467-023-43568-6)
Supplement: Supplementary file 3 — Description of Additional Supplementary Information [file 41467_2023_43568_MOESM3_ESM.docx]

**Description of Additional Supplementary Files**

Supplementary Data 1. Summary of sampling of the human developing cerebellum*.*

Supplementary Data 2. UMAP coordinate data of RNA-seq*.*

Supplementary Data 3. UMAP coordinate data of ATAC-seq*.*

Supplementary Data 4. DEGs of human developing cerebellum cell types*.*

Supplementary Data 5. Spatial-Specific Modules of the GW12 Human Cerebellum.

Supplementary Data 6. DEGs of RL Progenitor and VZ Progenitor*.*

Supplementary Data 7. DEGs of Progenitor subtypes*.*

Supplementary Data 8. Closing sites and opening sites of the Purkinje cell lineage*.*

Supplementary Data 9. Target genes of PTF1A and RORB*.*

Supplementary Data 10. Opening sites of the granule cell lineage*.*

Supplementary Data 11. DEGs of Early-Stage and Late-Stage Proliferating Granule Cells*.*

Supplementary Data 12. DEGs of Specific Genes in Humans.

Supplementary Data 13. DEGs of Specific Genes in Mouse.

Supplementary Data 14. Disease-associated genes in the cerebellum*.*

Supplementary Data 15. SNPs linked to the same gene with coding and non-coding region in the developing cerebellum.
