# Supplementary material for: Single-cell epigenomics and spatiotemporal transcriptomics reveal human cerebellar development
Source: Nat Commun. 2023 Nov 22;14:7613. doi: 10.1038/s41467-023-43568-6 (PMC10665552; doi:10.1038/s41467-023-43568-6)
Supplement: Supplementary file 19 — Reporting Summary [file 41467_2023_43568_MOESM19_ESM.pdf]

nature portfolio

Corresponding author(s): Qian Wu, Suijuan Zhong

Last updated by author(s): Nov 1, 2023

Reporting Summary

Nature Portfolio wishes to improve the reproducibility of the work that we publish. This form provides structure for consistency and transparency in reporting. For further information on Nature Portfolio policies, see our Editorial Policies and the Editorial Policy Checklist.

Statistics

For all statistical analyses, confirm that the following items are present in the figure legend, table legend, main text, or Methods section.

n/a	Confirmed	
☐	☒	The exact sample size (*n*) for each experimental group/condition, given as a discrete number and unit of measurement
☐	☒	A statement on whether measurements were taken from distinct samples or whether the same sample was measured repeatedly
☐	☒	The statistical test(s) used AND whether they are one- or two-sided<br>*Only common tests should be described solely by name; describe more complex techniques in the Methods section.*
☒	☐	A description of all covariates tested
☐	☒	A description of any assumptions or corrections, such as tests of normality and adjustment for multiple comparisons
☐	☒	A full description of the statistical parameters including central tendency (e.g. means) or other basic estimates (e.g. regression coefficient) AND variation (e.g. standard deviation) or associated estimates of uncertainty (e.g. confidence intervals)
☐	☒	For null hypothesis testing, the test statistic (e.g. *F*, *t*, *r*) with confidence intervals, effect sizes, degrees of freedom and *P* value noted<br>*Give P values as exact values whenever suitable.*
☒	☐	For Bayesian analysis, information on the choice of priors and Markov chain Monte Carlo settings
☒	☐	For hierarchical and complex designs, identification of the appropriate level for tests and full reporting of outcomes
☒	☐	Estimates of effect sizes (e.g. Cohen's *d*, Pearson's *r*), indicating how they were calculated

Our web collection on statistics for biologists contains articles on many of the points above.

Software and code

Policy information about availability of computer code

Data collection	The sequencing data was collected on the Illumina platform.
Data analysis	No custom software was used. Cellranger 2.1.1,Spaceranger (version 1.3.0), Python 3.7.2, Python package: Scanpy(v1.4.2),pySCENIC(v0.10.3), Scvelo (v0.2.2), R3.4.2, R package: Monocle2(v 2.6.4), Monocle3(v0.2.3), Seurat (v3.2.4),Signac(v0.2.5), pheatmap(v1.0.12),batchelor (v1.2.4),ggplot2 (v3.3.0) , riverplot (v0.6), SnapATAC (v 1.0.0) were used. See Methods for details on how each software is used.

For manuscripts utilizing custom algorithms or software that are central to the research but not yet described in published literature, software must be made available to editors and reviewers. We strongly encourage code deposition in a community repository (e.g. GitHub). See the Nature Portfolio guidelines for submitting code & software for further information.

Data

Policy information about availability of data

All manuscripts must include a data availability statement. This statement should provide the following information, where applicable:

- Accession codes, unique identifiers, or web links for publicly available datasets
- A description of any restrictions on data availability
- For clinical datasets or third party data, please ensure that the statement adheres to our policy

The scRNA-seq data and ATAC-seq data generated in this study have been deposited in the Gene Expression Omnibus (GEO) database under accession code GSE165657 [https://www.ncbi.nlm.nih.gov/geo/query/acc.cgi?acc=GSE165657] and in the Genome Sequence Archive (GSA) under accession number HRA000780

[https://ngdc.cncb.ac.cn/search/?dbId=hra&q=HRA000780]. The disease risk genes used in this study are available in the SFARI database [https://gene-archive.sfari.org/database/gene-scoring/] and the ClinVar database [https://ftp.ncbi.nlm.nih.gov/pub/clinvar/vcf_GRCh38/clinvar_20201219.vcf.gz]. Raw image files used in the figures that support the findings of this study are available from the corresponding authors upon request.

Research involving human participants, their data, or biological material

Policy information about studies with human participants or human data. See also policy information about sex, gender (identity/presentation), and sexual orientation and race, ethnicity and racism.

Reporting on sex and gender	All embryo and fetal tissued were between 12-27 gestational weeks. The sex and gender information of embryos was in supplemental table 1:Sheet 1: Summary of sampling of the human developing cerebellum. Both male and female samples were included.
Reporting on race, ethnicity, or other socially relevant groupings	Not relevant to this study.
Population characteristics	All embryo and fetal tissued were between 12-27 gestational weeks. Gestational age was measured in weeks from the first day of the woman's last menstrual cycle to the sample collecting date.
Recruitment	Beijing Anzhen Hospital was in charge of recruiting donors for this research. This study recruit the pregnant women in gestational weeks 12-27, who have decided to do the abortion under their personal willingness. No selection bias was present. The de-identified human fetal tissue samples were collected after the donor patients signing informed consent document.
Ethics oversight	The de-identified human tissue collection and research protocols were approved by the Reproductive Study Ethics Committee of Beijing Anzhen Hospital (2014012X) and the institutional review board (ethics committee) of the Institute of Biophysics(H-W-20170110).

Note that full information on the approval of the study protocol must also be provided in the manuscript.

Field-specific reporting

Please select the one below that is the best fit for your research. If you are not sure, read the appropriate sections before making your selection.

☒ Life sciences ☐ Behavioural & social sciences ☐ Ecological, evolutionary & environmental sciences

For a reference copy of the document with all sections, see nature.com/documents/nr-reporting-summary-flat.pdf

Life sciences study design

All studies must disclose on these points even when the disclosure is negative.

Sample size	No sample size calculation was performed. The sample size of scRNA-seq and snATAC-seq were determined by availability of human tissues. We collected 15 cerebellum from embryonic stages for scRNA-seq with 5 groups at the same developmental stage as replications. We collected 9 cerebellum from embryonic stages for snATAC-seq with 2 groups at the same developmental stage as replications. Final dataset scale was determined according to the quality control criteria as described in the methods.
Data exclusions	Only cells that expressed more than 800 genes and less than 5000 genes were considered, and only genes expressed in at least 3 single cells were included for further analysis by Seurat (Ver.3.2.4).
Replication	As scRNA-seq, 3 biological replicates in GW12, 2 biological replicates in GW14, 2 biological replicates in GW16, 2 biological replicates in GW18, 4 biological replicates in GW21. 3 replicates in GW12, 2 replicates in GW18 were used in scATAC-seq experiments. For animal experiments, at least 3 individual groups of experiments were performed. All attempts at replication were successful.
Randomization	The samples were allocated into each experimental groups based on the gestational stage. See methods for details.
Blinding	The investigators were blinded to group allocation during data collection and analysis.

Reporting for specific materials, systems and methods

We require information from authors about some types of materials, experimental systems and methods used in many studies. Here, indicate whether each material, system or method listed is relevant to your study. If you are not sure if a list item applies to your research, read the appropriate section before selecting a response.

Materials & experimental systems

n/a	Involved in the study	
☐	☒	Antibodies
☒	☐	Eukaryotic cell lines
☒	☐	Palaeontology and archaeology
☐	☒	Animals and other organisms
☒	☐	Clinical data
☒	☐	Dual use research of concern
☒	☐	Plants

Methods

n/a	Involved in the study	
☒	☐	ChIP-seq
☒	☐	Flow cytometry
☒	☐	MRI-based neuroimaging

Antibodies

Antibodies used	For immunostaining, the following antibodies to the following proteins were used:<br>Rabbit monoclonal [EPR19592] to MASH1/Achaete-scute homolog 1, Abcam, ab213151 ,GR33165-1;<br>Rabbit Polyclonal to PAX6, BioLegend, 901301, B201255;<br>Rabbit Polyclonal to TTYH1, Sigma, HPA023617, R10755;<br>Mouse monoclonal [CL-300] to Calbindin, Abcam, ab9481, 335124;<br>Rabbit Polyclonal to RORB, Invitrogen, PA5-28742, WA3163718B.
Validation	For primary antibody, we validated in human tissues for Immunofluorescence (IF) staining on frozen tissues. For methods detail, please see method section.<br>The manufacturer information regarding to the reactive species as follows:<br>Rabbit monoclonal [EPR19592] to MASH1/Achaete-scute homolog 1, Abcam, ab213151, for Human and Mouse;<br>Rabbit Polyclonal to PAX6, BioLegend, 901301, B201255, for Human, Mouse, Rat;<br>Rabbit Polyclonal to TTYH1, Sigma, HPA023617, R10755, for Human;<br>Mouse monoclonal [CL-300] to Calbindin, Abcam, ab9481, 335124 for Mouse, Rat, Sheep, Rabbit, Chicken, Guinea pig, Hamster, Human, Fish, Monkey;<br>Rabbit Polyclonal to RORB, Invitrogen, PA5-28742, WA3163718B, for Human, Mouse.

Animals and other research organisms

Policy information about studies involving animals; ARRIVE guidelines recommended for reporting animal research, and Sex and Gender in Research

Laboratory animals	Wildtype CD-1 mice which were timed pregnant at E11.5 were purchased from Charles River Laboratories in China. All mice had free access to food and water, were housed in the institutional animal care facility with a 12 h light-dark schedule. The humidity was kept at 50- 65%. Rooms and cages were kept at a temperature range of 20-26 oC. All the subjects were not involved in any previous procedures.
Wild animals	This study did not involved the wild animals.
Reporting on sex	The pregnant mice were female. The information of mice embryos were not collected. No sex was specifically selected.
Field-collected samples	This study did not involved the samples collected from the field.
Ethics oversight	Animal housing and all experimental procedures in this study were in compliance with the guidelines of the Institutional Animal Care and Use Committee of the Beijing Normal University (IACUC(BNU)-NKLCNL2019-09).

Note that full information on the approval of the study protocol must also be provided in the manuscript.
